# Optimization of cell-laden bioinks for 3D bioprinting and efficient infection with influenza A virus

**DOI:** 10.1038/s41598-018-31880-x

**Published:** 2018-09-17

**Authors:** Johanna Berg, Thomas Hiller, Maya S. Kissner, Taimoor H. Qazi, Georg N. Duda, Andreas C. Hocke, Stefan Hippenstiel, Laura Elomaa, Marie Weinhart, Christoph Fahrenson, Jens Kurreck

**Affiliations:** 1 Institute of Biotechnology, Department of Applied Biochemistry, Technische Universität Berlin, 13355 Berlin, Germany; 2 Berlin-Brandenburg Center for Regenerative Therapies & Berlin-Brandenburg School for Regenerative Therapies, Charité Universitätsmedizin Berlin, 13353 Berlin, Germany; 3 Department of Internal Medicine/Infectious and Respiratory Diseases, Charité – Universitätsmedizin Berlin, corporate member of Freie Universität Berlin, Humboldt-Universität zu Berlin, and Berlin Institute of Health, Germany, 10115 Berlin, Germany; 4 Institute of Chemistry and Biochemistry, Department of Organic Chemistry, Freie Universität Berlin, 14195 Berlin, Germany; 5 Center for electron microscopy (ZELMI), Technische Universität Berlin, 10623 Berlin, Germany

## Abstract

Bioprinting is a new technology, which arranges cells with high spatial resolution, but its potential to create models for viral infection studies has not yet been fully realized. The present study describes the optimization of a bioink composition for extrusion printing. The bioinks were biophysically characterized by rheological and electron micrographic measurements. Hydrogels consisting of alginate, gelatin and Matrigel were used to provide a scaffold for a 3D arrangement of human alveolar A549 cells. A blend containing 20% Matrigel provided the optimal conditions for spatial distribution and viability of the printed cells. Infection of the 3D model with a seasonal influenza A strain resulted in widespread distribution of the virus and a clustered infection pattern that is also observed in the natural lung but not in two-dimensional (2D) cell culture, which demonstrates the advantage of 3D printed constructs over conventional culture conditions. The bioink supported viral replication and proinflammatory interferon release of the infected cells. We consider our strategy to be paradigmatic for the generation of humanized 3D tissue models by bioprinting to study infections and develop new antiviral strategies.

## Introduction

Influenza A virus (IAV) is one of the most common causes of acute severe respiratory diseases worldwide. IAV infections are associated with high morbidity and mortality rates and have substantial socioeconomic impact^1,2^. Rodent models are widely used to study human lung diseases; however, these models suffer from severe limitations. According to a recent study, approximately 80% of potentially therapeutic drugs assessed effective in animals fail in humans^3^. An important problem is that mice in general are not natural hosts of IAV and are not susceptible to infection^4,5^. The majority of the known IAV strains replicate poorly in the murine respiratory tract and have to be adapted by serial passaging^6^. However, even adapted IAV strains can cause inconsistent outcomes of infection in different mouse strains, and the course of disease differs between humans and rodents^7^.

Tissue engineering approaches provide an option to overcome these shortcomings and help to minimize the gap between the different species. Within the last decade, the field of respiratory tissue engineering has advanced significantly^8,9^. Initially, approaches were developed to mimic the human pulmonary tract by conventional two-dimensional (2D) mono-cultures^10^. However, in conventional 2D culture systems, cells adhere to a flat surface so that the physiological status of the cells usually differs from the *in vivo* situation^11^. In addition, while IAV infection of the human respiratory tract does not homogenously effect every alveolar cell throughout the whole alveolar compartment, infection of 2D cultured monolayers is homogenous. To better mimic the spatial distribution of cells, the natural patterns of infection as well as cell-cell and cell-matrix interactions, sophisticated three-dimensional (3D) constructs consisting of a scaffold and various cell types have been developed^9,12^. These culturing conditions were found to positively impact proliferation, differentiation, survival and bioactivity of the cells^11,13,14^.

An up and coming strategy for tissue engineering is the use of 3D bioprinting technologies. The integration of living cells into bioactive materials which mimic components of the extracellular matrix (ECM) can generate 3D *in vitro* models that will contribute to our understanding of physiological mechanisms^15,16^. The development of models for investigating human-based pathologies of cardiovascular, cancer, skin and hepatotoxic diseases as well as for the development of novel therapeutics^17,18^ is supported by basic research on the interactions between biomaterials and cells^19,20^. Layer-by-layer deposition of bioinks allows controlled spatial positioning of cells, thereby facilitating the generation of precise and scalable structures, which 2D and standard 3D cell cultures cannot provide.

However, the complex production processes of 3D bioprinting are accompanied by various challenges, including limiting the mechanical stress during printing, adequate supply of the cells with nutrients during cultivation and the need for biocompatible materials^18,21–23^. Major requirements for the used bioinks are printability, biocompatibility and the support of structural and mechanical properties^24–26^. To meet these demands, microextrusion-based printing technologies often apply hydrogels, which maintain a steady state character due to a cross-linked polymer network within the fluid^27^. This technology allows the uninterrupted extrusion of bioinks within a broad viscosity range and provides spatial resolution high enough to generate geometrically complex tissue constructs^28–30^.

One of the most frequently used materials for microextrusion printing is alginate, a naturally occurring, polyanionic linear polysaccharide obtained from brown algae^31,32^. It is composed of (1–4)-linked β-D-mannuronic (M) and α-L-guluronic acids (G), which are ordered in mannuronic or guluronic blocks, separated by regions in which both acids are mixed. Cross-linking occurs rapidly between the G-blocks of adjacent polymer strand in the presence of divalent cations^27,31^. Alginate is characterized as a biocompatible material that does not intensively interact with cellular surfaces and whose negative charges enable interactions with positive charged ionic groups^33^. Water and smaller molecules can be trapped in the alginate matrix, but are still able to diffuse, thereby providing sufficient supply with nutrients^27^. Printability of alginate-based bioinks depends on their viscosity. Cations such as Ca^2+^ induce rapid gelation of alginate^34^. However, if the viscosity is too high during the extrusion process high pressure must be applied and the resulting mechanical forces and shear stress may damage the cells. On the other hand, low viscosity and slow gelation hamper structural reproducibility and resolution of the printed model.

The properties of bioinks can be improved by blending different biopolymers with distinct characteristics. Such mixtures can be used to combine the required printability and structural stiffness with high cell viability and metabolic activity of the cells. Frequently, blends of alginate and gelatin are used for extrusion-based bioprinting to combine the thermo-sensitive properties of gelatin^35^ with the chemical cross-linking capabilities of alginate. Gelatin provides good flow characteristics of the blended bioink during the printing process. As its temperature-induced gelation is faster than the Ca^2+^-induced gelation of alginate, it improves the initial stability of the printed construct. During subsequent cultivation of the 3D printed cell-laden constructs in culture media at a temperature above room temperature, the gelatin dissolves, whereas the cross-linked alginate maintains the structural integrity. Under the standard culturing conditions at 37 °C, gelatin completely dissolves and only alginate remains^36,37^.

While alginate provides desirable biocompatibility and high mechanical stability, it has poor biomimetic properties, since it does not provide cellular adhesion motifs due to the lack of essential protein components. In addition, mammalian cells cannot utilize alginate in their ECM^27^. To overcome these shortcomings, protein-based hydrogels have been introduced that provide necessary peptides, as well as growth factors, thereby facilitating adhesion and cell growth^38^. A prominent example is Matrigel, a gelatinous protein mixture secreted by Engelbreth-Holm-Swarm (EHS) mouse sarcoma cells^39^. As Matrigel itself lacks mechanical properties required during the bioprinting process, the creation of multicomponent bioinks combines the advantageous properties of different polymers and helps to generate 3D tissue models by microextrusion bioprinting^40^.

The aim of the present study was to generate a 3D tissue model for infection experiments. We have recently shown that a recellularized biological ECM can provide a suitable system to study the distribution of adeno-associated virus (AAV) vectors^41,42^. Here, we go one step further and optimize the composition of 3D printable cell-laden hydrogels to generate a suitable model for IAV infection. The blends were used to print human alveolar epithelial (A549) cells in a spatially controlled manner. The influence of variable amounts of growth factors and proteins in the 3D printed scaffold on the viability of the A549 cells was investigated to define requirements for long term culture. Different blends of alginate, gelatin, and Matrigel were characterized by biophysical methods and their suitability to support viability and metabolic activity of A549 cells was investigated. Finally, the bioprinted 3D models were infected with a naturally occurring seasonal IAV strain and spatial distribution of infected cells in different bioinks was analysed. While A549 cells can only be cultivated for approximately three days in 2D culture before they overgrow and start to change their phenotype, we found that our optimized model not only facilitates prolonged cultivation periods of the alveolar cells, but also provides a 3D model for influenza infection, including the induction of an immune reaction in A549 cells. We consider our approach paradigmatic for generating tissue models by bioprinting to study viruses and their interaction with host cells in a 3D environment.

## Results and Discussion

### Bioink development

**Figure 1:**
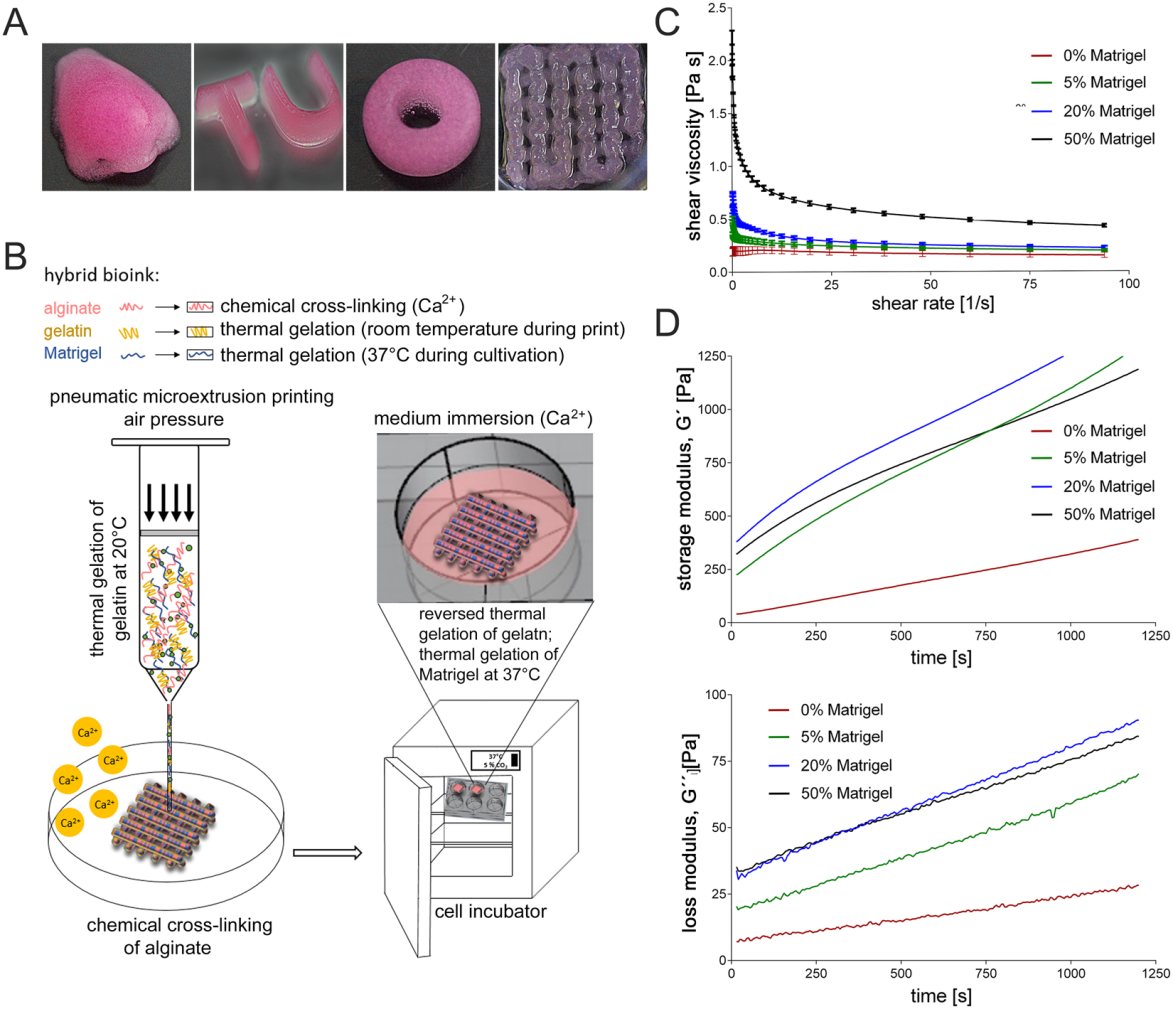
Experimental design with images of 3D printed constructs and rheological characterization of hybrid alginate/gelatin/Matrigel bioinks. (**A**) Image of constructs of different shapes and complexity generated by extrusion printing with the bioink optimized in the present study, consisting of 2% alginate, 3% gelatin, and 20% Matrigel. (**B**) Schematic workflow of the 3D printing procedure. Bioink components and living A549 cells were mixed thoroughly with two syringes connected by a Luer-Lock-adapter. Following initial Ca^2+^-driven cross-linking, the hydrogel was transferred into the dispensing cartridge in the print head and pneumatically extruded onto a dry petri dish. Thermal gelation of gelatin maintained the 3D structure of the construct during the printing process. Alginate was completely cross-linked by submersion in a CaCl_2_ solution. During incubation at 37 °C the gelatin dissolves, while the Matrigel gelation takes over support of the structural stability. (**C**) Viscosity of the uncross-linked hybrid bioinks was recorded dependent on a shear rate sweep of 1–100 s^−1^ at 24 °C. (**D**) Storage and loss modulus (G′ and G″) of the bioink formulations were measured at frequencies of 1 Hz and 1% shear strain after adding CaSO_4_ to determine viscoelastic properties of the cross-linked bioinks at 24 °C.

In initial experiments, we systematically optimized the bioink composition and cross-linking conditions to obtain a 3D construct for infection studies. A proportion of 2% (w/v) alginate is widely used for 3D scaffolds^43^. We tested various bioink compositions that contained, in addition to alginate, 1–10% (w/v) gelatin (data not shown). While a low concentration of gelatin was not sufficient to maintain the 3D shape fidelity during the printing process, too much resulted in hydrogels that were no longer printable. Based on these initial experiments, our basic bioink consisted of 2% (w/v) alginate and 3% (w/v) gelatin to enable extrusion printing at reasonable pressure and preserve 3D structural integrity during the procedure. Likewise, different concentrations of the ionic cross-linker CaSO_4_ were tested and 2.5% (w/v) of a 1.22 M solution was found to give the best results. In preparation for the printing process, the alginate/gelatin blend was heated to 37 °C and loaded into a syringe, while 1.22 M CaSO_4_ (2,5% w/v) were loaded into a second syringe. Both syringes were connected via a Luer-Lock adapter and the contents were thoroughly mixed. After 8 min at room temperature, the bioink was transferred into a printing cartridge. The pneumatic extrusion printer INKREDIBLE+ (Cellink) was used to generate patterns of various complexity, height and shape to determine the range of printable height and minimal spacing (Fig. 1A).

As human primary type II alveolar cells are difficult to obtain and can only be cultivated for a limited time before transdifferentiating into type I cells, we chose to include the A549 cell line in the hydrogel. This cell line is easy to expand and robust toward mechanical stress during the printing procedure. Although, being a tumour cell line, A549 cells were reported to be a suitable substitute for type II alveolar cells, as they resemble the primary cells with respect to metabolism, morphology, and ultrastructure^44^. In our cell-based approaches, we added 7 × 10^6^ cells/ml A549 cells and the indicated amount of Matrigel to the second syringe prior to mixing. All experiments with cell-laden bioinks were carried out with a construct with a rectangular pattern (length 1 cm × width 1 cm × height 0,1 cm), in which regularly spaced pores were laid out in a grid-like pattern. This pattern is widely used in 3D bioprinting applications, as the pores facilitate supply of the cells with media, nutrients and oxygen during cultivation, which is not achieved in organoids or in simple cast 3D constructs. The experimental flow chart is schematically illustrated in Fig. 1B.

To improve biocompatibility and attachment of the cells to the matrix, 5, 20 and 50% Matrigel, respectively, were added to the basic alginate/gelatin blend. Gelatin and Matrigel are thermo-sensitive materials. While gelatin gels at temperatures below 30–35 °C, Matrigel solidifies at temperatures above 4 °C. When mixing chilled Matrigel with the alginate/gelatin blend of 37 °C, Matrigel partially gels and accelerates the gel formation.

### Rheological characterization of different bioink compositions

Rheological measurements were carried out to characterize the properties of the different blends as rheological characteristics of the bioinks such as viscosity will influence their printability. First, viscosity of the temperature-dependent solidification of gelatin in different bioinks without chemical cross-linking of alginate was investigated. Cell-free solutions of alginate/gelatin and the respective volumetric fractions of Matrigel (0, 5, 20, and 50%) were thoroughly mixed and viscosity of the uncross-linked hybrid bioinks were recorded dependent on a shear rate sweep (Fig. 1C). Initial viscosity values increased with rising Matrigel concentration. All blends showed a strong decrease in their viscosity already at shear rates between 0 and 20 s^−1^, but remained nearly constant at higher shear rates.

Next, time-dependent changes of elastic and viscous properties of the hybrid inks were measured after addition of CaSO_4_ (2.5% w/v). All bioink formulations showed a vigorous increase of the storage modulus (G′) within 20 min (Fig. 1D). This effect was even more pronounced in Matrigel-blended bioinks. In contrast, the loss modulus (G″) only increased slowly, indicating that the hybrid hydrogels became stiffer due to the Ca^2+^-driven cross-linking of alginate. The time frame of 20 min was chosen based on the time needed for mixing the ink components, the CaSO_4_-induced initial cross-linking of alginate and the printing process itself. Increasing the Matrigel concentration from 0% to 5% resulted in a steep increase of the storage modulus. Raising the concentration further from 5% to 20% Matrigel only lead to a slight additional increase of G′. Interestingly, the storage modulus decreased when increasing the Matrigel content further to 50%. The values even dropped below those of the blend containing 5% Matrigel after approximately 12 min of cross-linking. In a recently published study, Fan *et al*. also recognized the compromising effects of too high Matrigel concentrations on the elastic properties of agarose/Matrigel blends^45^. Thus, the addition of high concentrations of Matrigel can be expected to decrease the elastic properties of the alginate/gelatin bioink blend. In the study of Fan *et al*. the printability of the bioink containing 50% Matrigel was poor compared to bioinks blended with lower volumetric fractions of Matrigel, whereas in our experiments the printed structure was well preserved throughout the printing process in the presence of 50% Matrigel. However, while the structure remained stable during, as well as after, printing, the shape of the 3D models was easily damaged when transferring them into culture plates. Maintenance of the structural integrity required complete cross-linking of alginate induced by submerging the constructs in a solution of 100 mM CaCl_2_ for 5 min.

According to a recent study by Gao *et al*.^46^, the elastic and loss modulus values of the basic alginate (2% w/v)/gelatin (3% w/v) bioink used here were low enough to ensure high cell viability and extrudability. The tan(δ) values of our materials (0.05–0.2) were lower than suggested to provide optimal structural integrity of the alginate/gelatin material (0.25–0.45). However, the structural integrity was improved by blending the bioink with Matrigel. Higher concentrations of alginate and gelatin as used in the study by Gao *et al*. would have increased the viscosity and the elastic/loss modulus, but at the same time can be expected to impair A549 cell viability due to the requirement of higher pressure during the printing process.

### Characterization of cell distribution in cell-laden bioinks

**Figure 2:**
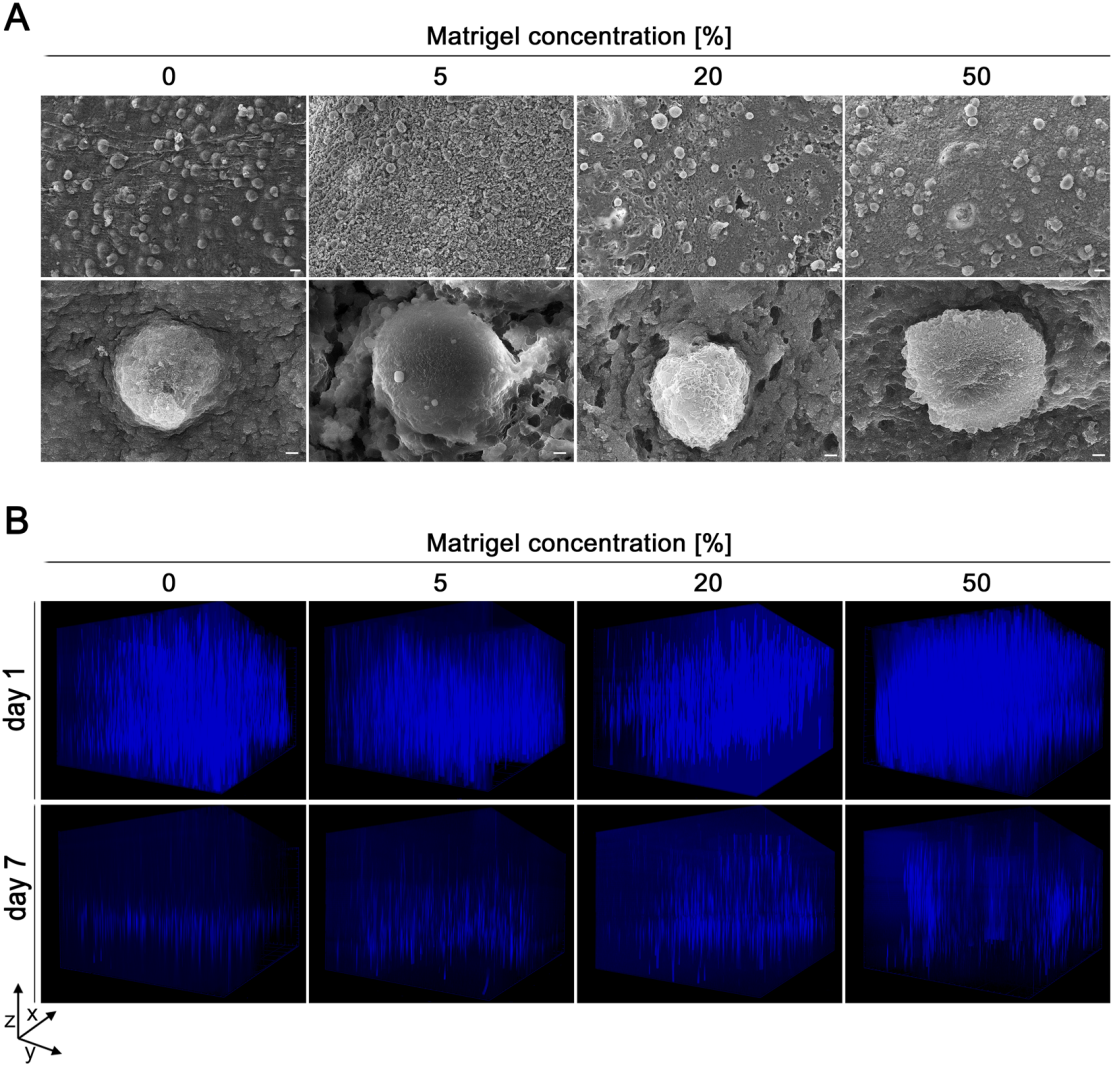
Representative SEM images of cell-laden constructs and fluorescence z-stack images of the spatial A549 cell distribution. (**A**) 3D printed cell-laden alginate/gelatin/Matrigel constructs were cultured for two days and processed for scanning electron microscopy. Each Matrigel concentration provided embedding of the cells within the constructs. Scale bars are indicated as 20 µm in the upper row and 1 µm in the lower one. (**B**) Three-dimensional distribution of A549 cells in 3D printed constructs one and seven days after printing visualized by nuclear Hoechst staining (blue) and Z-stack analysis from the top of the gel to the dish surface (scanning depth 1000 µm, interval 15.12 µm, area 1800 × 1400 µm). Figure B shows a representative image of three independent experiments.

Following the rheological characterization of cell-free bioinks, the cell distribution in the hydrogels was investigated. The microstructural surface morphology of the printed cell-laden hydrogels containing varying Matrigel concentrations was analysed by scanning electron microscopy (SEM). In general, the microstructure of all bioinks was homogenous, with a regularly spaced, even distribution of encapsulated A549 cells (Fig. 2A). Noticeably, the blends containing 5% and 20% Matrigel, respectively, showed a comparable degree of porosity with pore sizes of approximately 1–2 µm. In contrast, no porosity was observed in hydrogels without and with 50% of Matrigel. Porosity of the material is essential to support supply of the cells deeper in the printed construct with sufficient nutrients, thereby maintaining their viability.

The next step was to analyse the spatial distribution of A549 cell in the printed constructs by fluorescence microscopy. Nuclei were stained with Hoechst and 3D distribution was visualized by Z-stack analysis. After one day of culture, cells were well distributed in all bioinks under investigation, i.e. the Matrigel content did not influence the initial distribution of the cells (Fig. 2B, upper row). However, on day seven after the printing process, distinct differences became obvious (Fig. 2B, lower row). In the absence of Matrigel, cells sank to the bottom of the construct. This can easily be ascribed to the lack of cell-adhesive peptide motifs (e.g. RGD) that support attachment of the cells to the matrix as the molten gelatin was most likely washed out of the construct of the construct upon cultivation at 37 °C. The construct containing 5% Matrigel showed that not all cells sank, though many did. In contrast, higher Matrigel concentrations of 20 and 50% were substantially superior to support long-term spatial distribution of the cells than lower amounts of Matrigel. Thus, the higher Matrigel content supported cellular attachment to the matrix. In addition, it improved stability of the 3D printed constructs. Still, there was an obvious loss of the overall cell number with all bioink formulations used during seven days of culture, which was less prominent with the bioinks including higher Matrigel concentrations.

### Analysis of cell viability and cytotoxicity

**Figure 3:**
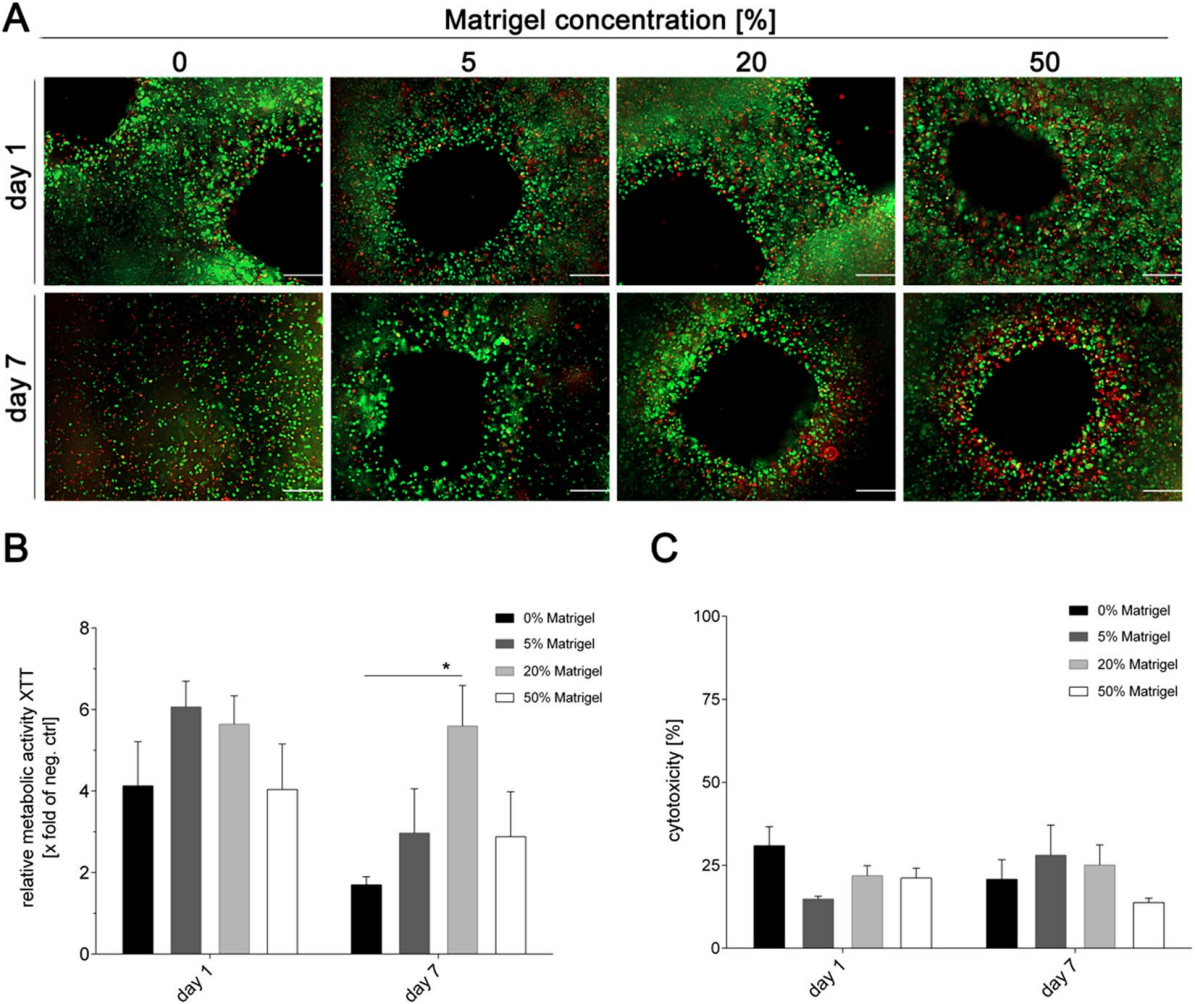
Viability of A459 cells in 3D printed alginate/gelatin constructs with varying Matrigel concentration. (**A**) Qualitative viability staining of living and dead A549 cells printed in the constructs after one and seven days of cultivation using calcein-AM (live in green) and ethidium homodimer-1 (dead in red). Scale bar: 200 µm. (**B**) Metabolic activity of A549 cells inside the different 3D printed alginate/gelatin/Matrigel constructs was determined by the XTT assay at the indicated time points. Values were calculated as X-fold induction of lysis control. (**C**) Cytotoxicity was analysed by the LDH assay. Data are depicted as percentage of the LDH amounts of cells in 3D printed constructs relative to the lysis control. Results are shown as mean ± *SEM* of three independent experiments. *p < 0,05 compared to 0% Matrigel.

Cell viability was qualitatively evaluated by microscopic analysis of living and dead cells on day one and seven of the culture. As can be seen in Fig. 3A, cell viability was high in all bioinks used on day one of culture. To quantify the fraction of viable cells, constructs were dissolved and ratios of living and dead cells were determined by trypan blue staining using a standard haemocytometer. Initially, the viability was slightly below 80% (±6.5%) for the bioinks containing 5 and 50% Matrigel, respectively, but was around 90% (±2.8%) in 0 and 20% Matrigel. On day seven of culture, the overall number of A549 cells decreased in all conditions, and the fraction of calcein-AM positive, i.e. living, cells was also slightly decreased. In the absence of Matrigel, viability dropped below 70% (±5.7%) and a substantial loss in cell number was observed. The fraction of viable cells was approximately 80% (±6.9%) in bioinks containing 5 and 20% Matrigel, respectively, while it dropped below 75% (±9.9%) in the construct printed with 50% Matrigel. Thus, a high Matrigel content of 50% maintains a high degree of spatial distribution over time (Fig. 2B), but despite the high content of proteins and growth factors, viability was decreased compared to bioinks with a lower Matrigel content. This is most likely due to the lower degree of porosity observed in the SEM analysis (Fig. 2A), which results in insufficient supply of the cells with nutrients. The same is true for the bioink without any Matrigel. In this case, the printed shape was also instable and macroscopic pores in the printed grids coalesced after 5–7 days in culture, which further limits the nutrient supply of the A549 cells located in the centre of the constructs and explains the lowest viability rate of all tested conditions.

The metabolic activity of the printed A549 cells was determined by measuring the reduction of the tetrazolium salt XTT to formazan by dehydrogenase enzymes on days one, three and seven after printing. Metabolic activity, i.e. viability of cells remained high until day three (data not shown), but substantially dropped on day seven of the culture under most conditions (Figure 3B). Only the bioink containing 20% Matrigel maintained significantly higher metabolic activity. These data are in line with the above described qualitative observations that the Matrigel content reaches an optimum at 20%, whereas a lower or higher concentration negatively impacts cell viability.

Finally, cytotoxicity resulting from the different inks was determined by measuring the release of lactate dehydrogenase (LDH). Figure 3C shows that cytotoxicity of all four bioinks was comparatively low (≤25% compared to the lysis control), and only a minor increase of about 5% was observed for the cultivation period of seven days, which is also typical for conventional 2D cell culture systems. No statistically significant differences were observed between the different Matrigel contents.

The stiffness of the 3D printed polymers directly depends on the amount of Matrigel included in the bioink. This reflects well the natural biomechanical properties of the lung, which are related to the stiffness of the ECM. Fibrillary proteins of the ECM maintain the tensile strength^47^, whereas elastin fibre components are responsible for elastic recoil^48^. The stiffness of different areas of the normal lung parenchyma varies and ranges from 0.44 to 0.75 kPa^49^. Even slight changes of protein expression levels or their ratios impacts the ECM stiffness and contributes to severe respiratory illnesses as reviewed in Burgess *et al*.^49^. In our experiments, the addition of 50% Matrigel did not alter the pressure required during the printing process, but following complete solidification of Matrigel at 37 °C, the stiffness of constructs containing 50% Matrigel was higher than that of bioinks with a lower Matrigel content. Together with the reduced porosity, this may explain the limited viability of alveolar epithelial cell, which are incorporated in highly elastic soft tissue in the native lung.

### Infection of cell-laden 3D printed biopolymers with influenza A virus

**Figure 4:**
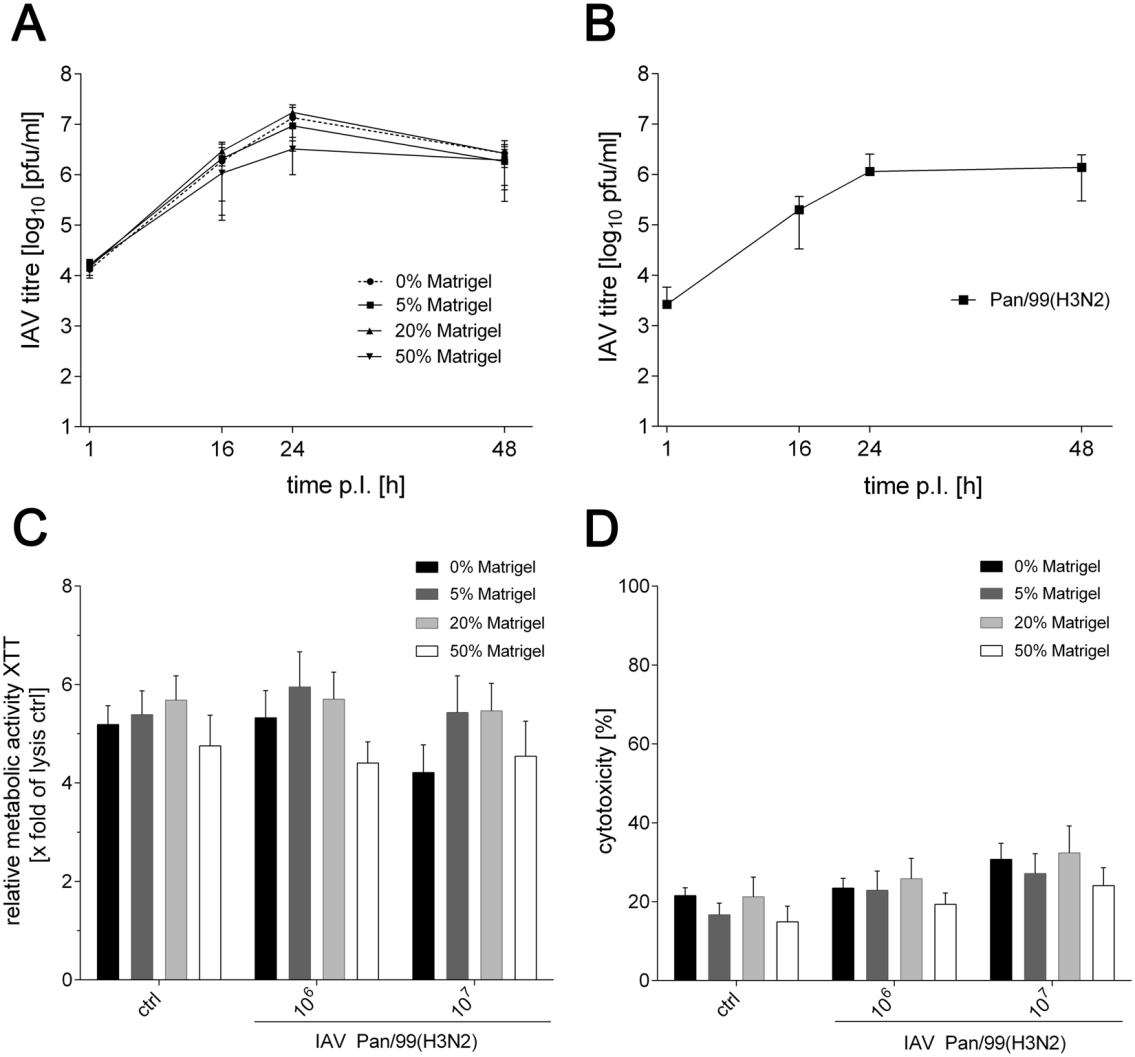
Influenza A virus replication in 3D printed and 2D cultured A549 cells. (**A**) Printed cell-laden alginate/gelatin constructs with varying Matrigel concentrations were infected with 4 × 10^5^ PFU of the Pan/99(H3N2) virus. Supernatants were collected at indicated time points and titrated on MDCK cells. (**B**) 2D cultured A549 monolayers were infected with the Pan/99(H3N2) virus at an MOI of 1, and collected supernatants were titrated on MDCK cells. (**C**) Metabolic activity of A549 cells was determined by XTT assays 24 h after infection of the 3D printed alginate/gelatin/Matrigel constructs with 10^6^ or 10^7^ PFU of Pan/99(H3N2). Values were calculated as X-fold induction of the lysis control. (**D**) Cytotoxicity resulting from infection with either 10^6^ or 10^7^ PFU of Pan/99(H3N2) was analysed by the LDH assay 24 h after infection. Data are depicted as percentage of the LDH amounts of cells in 3D printed constructs relative to the lysis control. Results are shown as mean ± *SEM* of three independent experiments.

To assess the suitability of the 3D printed models for infection experiments, we infected the cell-laden alginate/gelatin/Matrigel constructs with the prototypic seasonal influenza A virus strain A/Panama/2007/1999(H3N2) (Pan/99(H3N2)). Virus replication was systematically compared in A549 cells, either grown in conventional 2D culture or in 3D printed tissue models. The latter were infected one day after printing to allow recovery of the cells from the extrusion process. In both culture types, 2D and 3D, IAV productively infected A549 cells, as can be seen by the strong increase of infectious virus particles in the culture supernatants (Fig. 4A,B). For A549 cells cultured under 3D conditions, maximum viral titres of approximately 10^7^ plaque forming units (PFU)/ml were detected after 24 h of infection. The Matrigel content did not have a substantial influence on the replication efficiency, and only a minor, but statistically not significant, reduction of propagation efficiency was observed for 50% Matrigel after 24 h. As protease activity is required for cleavage activation of viral hemagglutinin, but was not provided by the 3D printed polymer backbone or Matrigel, even at the highest concentration used (data not shown), identical amounts of TPCK-trypsin were added in all cases to enable multicyclic Pan/99(H3N2) replication^50^. Based on the highest viral titres detected after 24 h of replication, all subsequent experiments were conducted with a PFU of 10^6^ or 10^7^. As each construct contains approximately 10^6^ cells, this equates to a multiplicity of infection (MOI) of 1 or 10, respectively.

After confirming proper Pan/99(H3N2) replication in the 3D constructs, metabolic activity of the printed A549 cells and cytotoxic effects following the infection were analysed. Reduction of the tetrazolium salt XTT and release of LDH were determined 24 h after infection of the tissue models with 10^6^ or 10^7^ PFU of the Pan/99(H3N2) virus. As can be seen in Fig. 4C, virus infection did not have a substantial effect on the metabolic activity. Only a small, but statistically not significant, decrease of XTT reduction was observed for the highest IAV dose in the absence of Matrigel.

LDH release, as a measure for cytotoxicity induced by IAV infection, was comparable in all bioinks (Fig. 4D). In the constructs containing 50% Matrigel, cytotoxicity was slightly lower compared to the other bioinks; however, the difference did not reach statistical significance. We observed a general tendency that cytotoxicity slightly increased in all formulations dependent on the virus dose, ranging from approximately 20% in uninfected samples to ~25% in response to IAV PFU of 10^6^ and close to 30% in models infected with an IAV PFU of 10^7^. As influenza virus infections can induce cell damage^51–53^ this slight increase in cytotoxicity is a reasonable pathogenic outcome of the infection, but is not expected to impair measurements of inflammatory parameters like cytokine or chemokine release.

**Figure 5:**
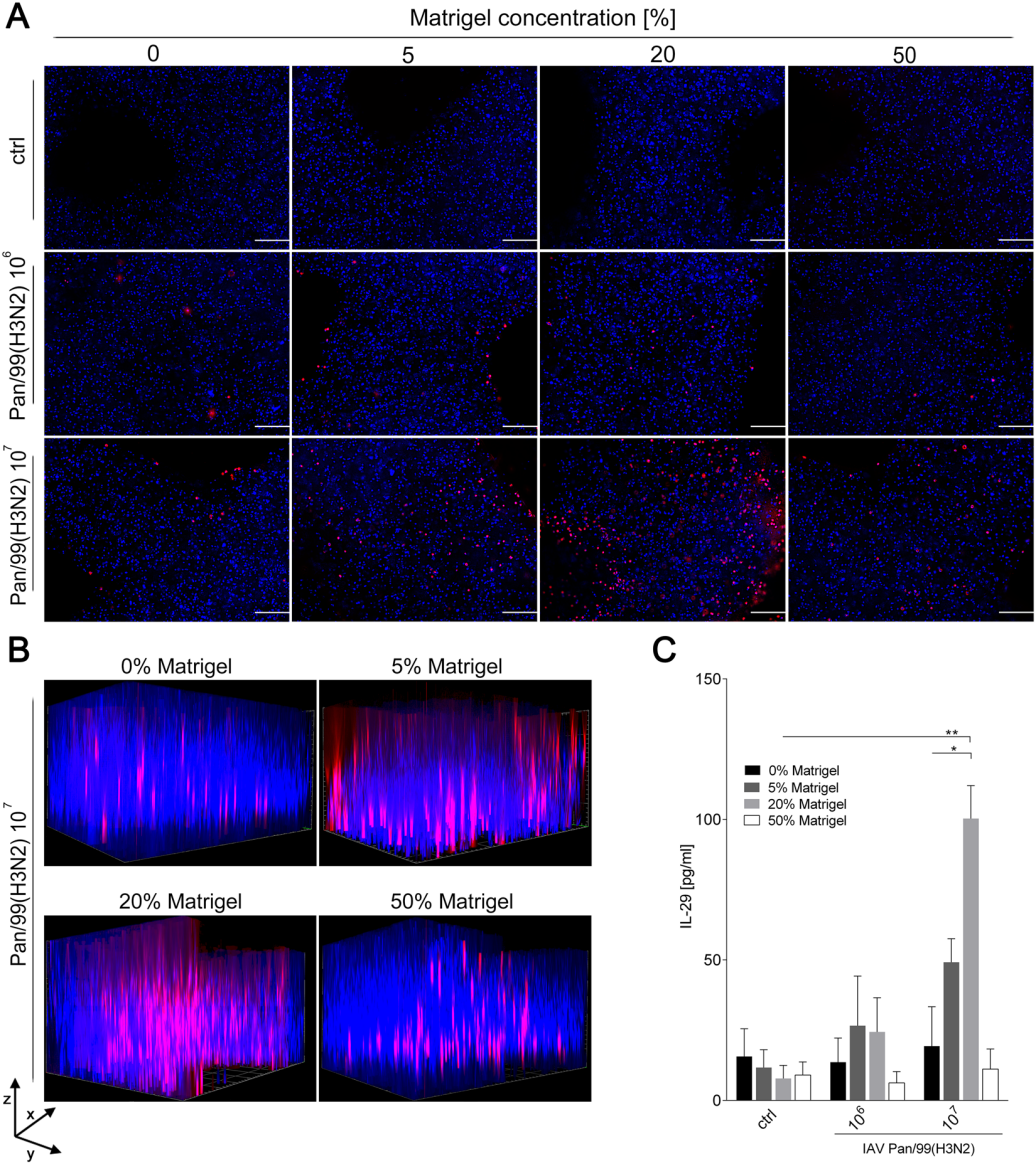
Distribution of Pan/99(H3N2) in cell-laden alginate/gelatin/Matrigel matrices and IL-29 release. 3D printed cell-laden alginate/gelatin constructs with varying Matrigel concentrations were infected with either 10^6^ or 10^7^ PFUs of Pan/99(H3N2) 24 hours after printing. (**A**,**B**) Fixed constructs were immunohistochemically labeled with anti-nucleoprotein antibody (red channel), and nuclear counter staining was performed using Hoechst stain (blue channel) 24 h after infection. Stained constructs were analysed by fluorescence microscopy by (**A**) top view (scale bar: 200 µm) or (**B**) Z-stack analysis to visualize spatial distribution of infected A549 cells (scanning depth 1000 µm, interval 15.12 µm, area 1800 × 1400 µm). (**C**) Supernatants of infected constructs were collected 24 h after Pan/99(H3N2) infection and release of IL-29 determined by ELISA. Results are shown as mean ± *SEM* of three independent experiments. *p < 0,05; **p < 0,01 compared to 0% Matrigel.

To visualize the efficiency and distribution of the IAV Pan/99(H3N2) infection within the 3D printed constructs, they were fixed 24 h post infection and stained for IAV nucleoprotein (NP). Nuclear counter staining was performed with Hoechst to depict the overall number of cells. All Matrigel concentrations showed NP-positive cells following infection with IAV with 10^6^ PFUs (Fig. 5A). The number of NP-positive cells was substantially increased after infection with the higher dose of 10^7^ PFUs. Interestingly, the fraction of IAV-positive cells differed depending on the bioink used. The number of infected A549 cells was higher in constructs containing 5% and 20% Matrigel compared to those without or with the highest Matrigel content of 50%. As cell viability was comparable at early time points for all conditions (see above) and cells were infected with the virus 24 h after printing, differences in cell viability are unlikely to account for improved infectivity in 5 and 20% Matrigel. The reasons for this finding need to be investigated in future experiments.

The spatial distribution of Pan/99(H3N2) viruses in the construct was determined by Z-stack analysis and revealed a cluster-like infection pattern (Fig. 5B). As for the top view, constructs with bioinks containing 5% or 20% Matrigel had the highest number of NP-positive cells. Important for the usability of the 3D tissue model was the random distribution of infection clusters throughout the construct rather than exclusive infection of the peripheral regions. This indicates that the grid structure with the pores as described above supports distribution of medium and viruses throughout the complete model. In addition, the clustered infection observed in our 3D construct reflects the natural situation in human lung tissue, where a similar phenomenon was found for several types of pathogens^54–56^.

The higher number of NP-positive cells observed by fluorescence microscopy in constructs composed of 5% and 20% Matrigel (Fig. 5) seems to contradict comparable Pan/99(H3N2) titres in replication assay for all conditions (Fig. 4A). A possible reason might be the absorption of small molecules like TPCK-trypsin by the polymer construct, resulting in limited cleavage activation of hemagglutinin. Thus, even a higher infection efficiency of A549 cells printed in the presence of 5% and 20% Matrigel would not result in higher virus titres as the virus sticks to the cells and cannot be released into the supernatant. The remaining amounts of TPCK-trypsin may still be sufficient to release a comparable number of viruses in the constructs that have a lower initial infection rate (i.e. 0% and 50% Matrigel). Follow-up experiments will be carried out with higher concentrations of TRCP-trypsin to test whether this would help to overcome limitation of virus replication caused by insufficient protease-mediated HA cleavage.

**Figure 6:**
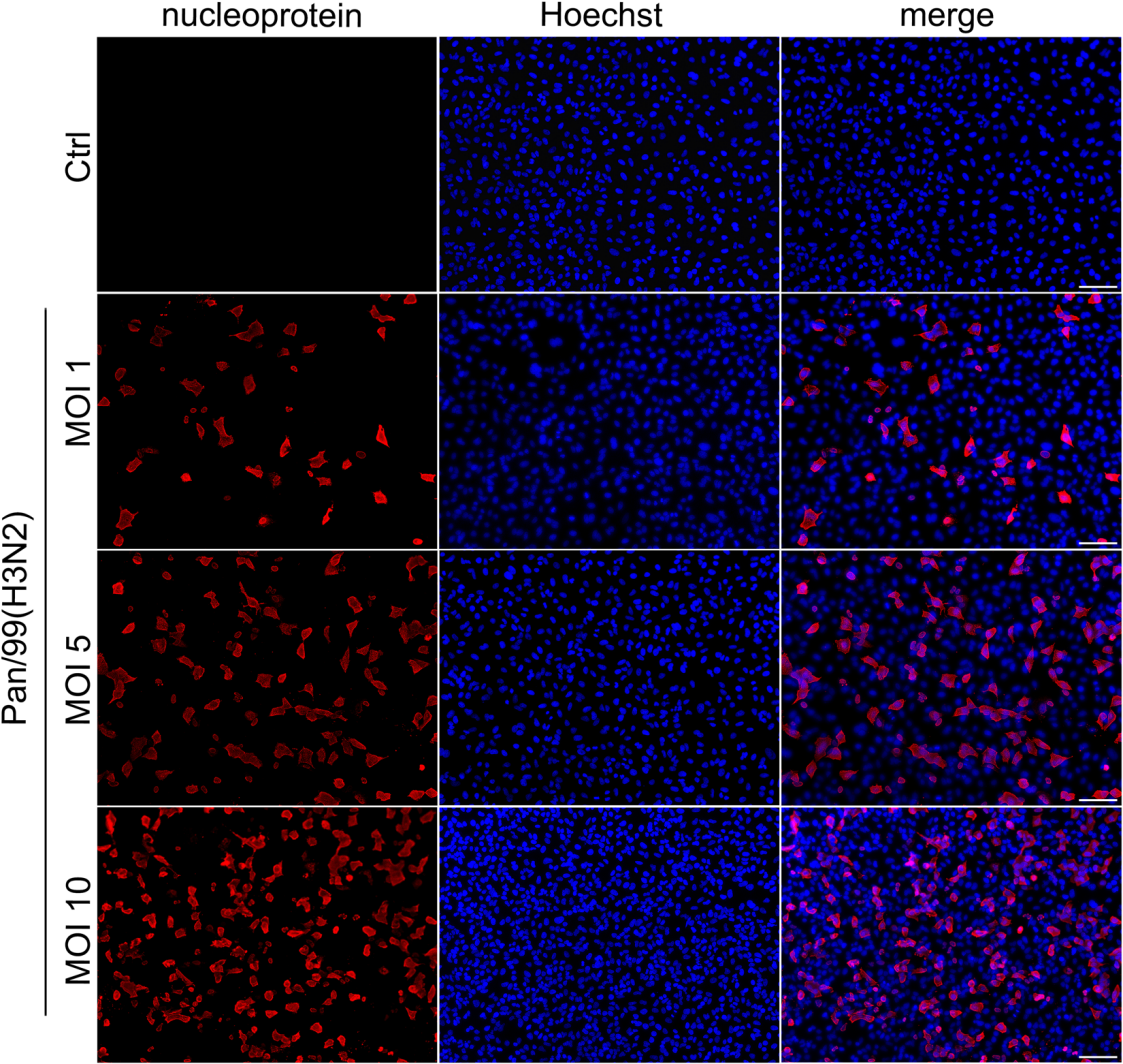
Infection pattern of IAV Pan/99(H3N2) in 2D cultured A549 cells. A549 cells were seeded in 24 well plates and infected with indicated MOIs of IAV Pan/99(H3N2) for 24 h. Fixed cells were immunohistochemically labelled with anti-nucleoprotein antibody (red channel), and nuclear counter staining was performed using Hoechst stain (blue channel). Stained A549 cells were analysed by fluorescence microscopy. Scale bar: 100 µm.

To compare features of 2D and 3D culture systems, we also carried out IAV infection experiments with 2D cultured A549 cells. As can be seen in Fig. 6, infection of A549 cells in 2D culture results in an even distribution of infection. As the clustered infection pattern observed in the 3D printed construct (Fig. 5B) more closely resembles the biological situation, this demonstrates the advantage of using sophisticated 3D cultures.

As a final experiment to test whether the IAV infection induces a cellular inflammatory response in 3D printed A549 cells, release of the antiviral interferon interleukin 29 (IL-29; also known as interferon λ_1;_ IFN_λ1_) was analysed^57^. In line with the limited infectivity in tissue models containing 0% and 50% Matrigel, no substantial IL-29 release was detected in the supernatant of these constructs following Pan/99(H3N2) infection (Fig. 5C). In contrast, IL-29 release correlating with the infectious virus dose was measured in the supernatant of cells printed with 5% and 20% Matrigel. Particularly for the latter conditions, A549 cells produced pronounced and statistically significant amounts of IL-29. Thus, the tissue models produced with cell-laden bioinks are capable of mimicking a basic immune response, in which the amount of IL-29 released correlates with the number of virus-infected cells according to microscopic analysis.

Despite the advantageous features of the model described here, it has some limitations in mimicking the natural situation. Most importantly, alveolar cells do not grow in a solid spatial arrangement, as is the case in the models presented here. Still, their natural growth differs from a flat 2D cultivation and the 3D interactions in printed constructs are important in order to study the biological function. Thus, neither 2D cultures, nor the 3D model presented here will reflect all aspects of alveolar cells *in vivo*, but by offering a 3D environment, spread can be assessed in a more realistic manner.

Several approaches are in progress to further improve the model. An important issue will be to replace the A549 cell line by primary cells. However, human type II ACEs are difficult to obtain and can only be maintained for a short time until they transdifferentiate into type I ACEs. We therefore established the model with the A549 cell line which is easy to cultivate and more tolerant of the mechanical stress imposed by the printing procedure. For successful 3D cultivation of primary ACEs, encapsulation of the cells by alginate is another issue that needs to addressed. While the encapsulation by alginate protects the cells during the printing procedure, it prevents cell-cell interaction during the subsequent experiments. This problem is common to most extruder-based bioprinting approaches reported to date. However, strategies to overcome this limitation have been proposed, e.g. the use of hydrogels that are sensitive to Matrix Metalloproteases (MMPS)^58^. Furthermore, experiments are ongoing to better mimic the natural situation by printing a 3D scaffold of fibroblasts on which a monolayer of alveolar cells can be grown. In addition, a dynamic component is planned to be introduced, as its effectiveness has been shown in the famous lung model introduced by the Ingber group^59^. Furthermore, we think that the model developed here is not only suitable to study influenza viruses, but can be regarded as a general strategy to investigate virus infection in solid organs.

## Conclusion

To the best of our knowledge, this study describes the first attempt to generate 3D bioprinted humanized models for infection studies with influenza virus. Cellular distribution, viability and infection efficiency were optimized by adjusting the Matrigel content in the printed bioink. We found a bioink composed of 2% alginate and 3% gelatin, supplemented with 20% Matrigel, to provide superior conditions for producing 3D lung models with A549 cells by bioprinting. The resulting tissue model supported widespread infection with IAV in clustered patterns that are also observed in the natural human lung. In addition, printed cells exhibited a basic immune response by releasing the antiviral IL-29 (interferon λ_1_). We envision that the model developed here is not only suitable to study influenza infections, but may also be used for other viruses and may support the development of new antiviral strategies in humanized *ex vivo* systems.

## Methods

### Cells culture and virus preparation

Human epithelial lung carcinoma cells (A549; ATCC, USA) were cultured using DMEM high glucose (Biowest, France) supplemented with 10% fetal bovine serum (FBS; c.c.pro, Germany), 1% 100X L-Glutamine (Biowest) and 1% pentamycin/streptomycin (P/S; Biowest). Madine Darby canine kidney (MDCK) cells were propagated with MEM (Thermo Fisher Scientific, USA) supplemented with 10% FBS, 1% 100x GlutaMax (Thermo Fisher Scientific) and 1% P/S. The seasonal human influenza A (H3N2) virus (A/Panama/2007/1999) was grown on MDCK cells. Virus stocks were aliquoted, stored at −80 °C and titrated on MDCK cells by plaque assays.

### Preparation of cell-laden biopolymers

Gelatin powder (Sigma, USA) was dissolved in DMEM high glucose on a magnetic stirrer at 1250 min^−1^ (37 °C). After 2 h, sodium alginate powder (Sigma, USA) was added under incessant stirring overnight (37 °C). The hybrid alginate (4.5% w/v) - gelatin (6.5% w/v) hydrogel was mixed with undiluted liquid Matrigel (Corning, USA) in various concentrations, A549 cells (7 * 10^6^ cells/ml), 1.22 M CaSO_4_ (Roth, Germany) and DMEM high glucose to obtain the final cell-laden hybrid alginate/gelatin/Matrigel bioink composed of alginate (2% w/v)/gelatin (3% w/v)/Matrigel (0; 5; 20 or 50% w/v)/2.5% CaSO_4_/7 * 10^6^ A549 cells/ml. Following CaSO_4_-driven initial cross-linking of alginate (8 min after mixing), the cell-laden hydrogel bioink was loaded into the cartridge.

### 3D Bioprinting

The INKREDIBLE+ microextrusion printer (Cellink, Sweden) was used for 3D bioprinting. Contamination was avoided by the HEPA filter system unit included in the printer. The bioink was extruded through a 22 G needle at 10–20 kPa to print a 3D construct, designed by the CAD software Rhinoceros5 (Robert McNeel & Associates, USA). The printed constructs were submerged in 100 mM CaCl_2_ (Roth) to increase gelation of alginate and subsequently cultured in an incubator at 37 °C and 5% CO_2_ in DMEM high glucose with supplements for up to 7 days.

### Rheological properties

Viscoelastic testing was performed using a Kinexus lab+ oscillating rheometer (Malvern, UK) with an active hood Peltier plate cartridge (Malvern, UK). Temperature was maintained at 24 °C during testing, representing printing conditions. Blended bioinks were prepared as mentioned above immediately prior to measurements, loaded on the plate, and the measuring geometry was lowered into position. Viscosity was recorded during a shear rate sweep of 1–100 s^−1^ within 2 min using a 40 mm, 2° cone plate geometry. The measurement was conducted without CaSO_4_. The viscoelastic regime of the bioinks was determined with an amplitude sweep from 0.1% to 50% strain. Storage and loss moduli (G′ and G″, respectively) were recorded at the frequency of 1 Hz and strain of 1% using an 8 mm parallel plate geometry within 20 min after addition of CaSO_4_.

### Scanning Electron Microscopy (SEM)

Sample preparation was performed as described by Hazrin-Chong and Manefield^60^. Briefly, constructs were fixed overnight in 2% glutaraldehyde (Sigma) (in 0.1 M cacodylate buffer (Sigma), pH 7.3) at room temperature. Fixed constructs were dehydrated in raising concentrations of ethanol (50-70-90-100-100%) and dried in hexamethyldisilazane for 5 min. Gold coating of 4 nm was done with a Balzers Union SCD 030. The surface morphology was determined with a GeminiSEM 500 microscope (Carl Zeiss, Germany).

### Cell viability and lactate dehydrogenase release

Metabolic activity of A549 cells printed in the alginate/gelatin/Matrigel bioink was determined using the tetrazolium hydroxide salt (XTT) assay according to the manufacturer’s instructions (AppliChem, Germany) at indicated time points. Briefly, printed cell-laden constructs (~10^6^ cells/construct) were cultured for seven days (37 °C, 5% CO_2_). XTT reagent (1 mg/ml) was added and incubated for 4 h. The absorbance of the resulting solution was measured spectrophotometrically at A450 nm (TriStar Multimode Reader LB942, Berthold Technologies, Germany) with a reference of A620 nm. Cell-laden constructs incubated in culture medium supplemented with 10% Triton-X-100 (ROTH, Germany) were used as lysis control. Values were normalized to lysis controls.

For the LIFE/DEAD assay (Viability/Cytotoxicity kit, Thermo Fisher Scientific, USA), 3D printed cell-laden constructs were stained with 2 µM calcein-AM and 4 mM ethidium homodimer-1 diluted in 1x HBSS (Thermo Fisher Scientific, USA) for 30 min (37 °C, 5% CO_2_). The samples were analysed by fluorescence microscopy (Zeiss Observer. Z1 microscope; Zeiss, Germany).

To quantify the fraction of viable cells, constructs were dissolved in 0.9% NaCl (Sigma) containing 55 mM Sodium acetate (Sigma) and 20 mM EDTA (Sigma). After centrifugation (300 × g, 3 min), the cell pellet was resuspended in 1x PBS, and ratios of living and dead cells were counted using a standard haemocytometer after trypan blue staining (BioRad, Germany).

Lactate dehydrogenase (LDH) release in the supernatant was determined at an absorbance of A492 nm with a reference of A620 nm (Sunrise absorbance microplate reader, Tecan, Switzerland) using a LDH detection kit (Roche, Switzerland).

### Cell distribution and immunofluorescence staining

For cellular distribution studies, 3D printed constructs were fixed in 4% formaldehyde (Sigma) and permeabilised with 1% Triton-X-100 for 15 min. Subsequently, nuclear staining was performed with 1 µg/ml Hoechst (H33342, AppliChem, Germany) for 1 h at room temperature.

For immunofluorescence staining, constructs were fixed and permeabilised as described above. Afterwards, constructs were blocked with 5% goat serum (Sigma) for 30 min and incubated with diluted anti-influenza NP antibody (ab20343, Abcam, UK, 1:1,000) overnight at 4 °C. Afterward, constructs were incubated with Alexa Fluor 594-conjugated goat-anti-mouse secondary antibody (A11005, Thermo Fisher Scientific, USA; 1:2,000) overnight at 4 °C. Nuclear counterstaining was performed using Hoechst stain (1 µg/ml) for 1 h at room temperature. Cellular distribution and immunofluorescence were analysed with the Zeiss Observer. Z1 microscope (Zeiss, Germany).

### Influenza infection

For infection experiments, cell-laden 3D printed construct were washed three times with 1x HBSS and inoculated with Pan/99(H3N2) (10^6^ or 10^7^ PFU) for 1.5 h at room temperature. Afterwards the solution was removed, washed once with 1x HBSS and replaced by DMEM high glucose supplemented with 2% FBS, 1% 100X L-Glutamine, 1% P/S and 0.2 mg/ml TPCK-trypsin (Thermo Fisher Scientific, USA). Samples were incubated for 24 h (37 °C; 5% CO_2_) and prepared for further analysis.

### Interleukin 29 secretion

Concentrations of human interleukin 29 (IL-29/IFN-λ_1_) in supernatants of infected cell-laden 3D printed constructs were determined with the IL-29 Human ELISA Kit (Thermo Fisher Scientific, USA) at A450 nm (TriStar Multimode Reader LB942, Berthold Technologies, Germany) with a reference of A620 nm.

### Plaque assay

For growth curve analysis of Pan/99(H3N2), inoculation of 3D printed cell-laden constructs was performed with 4 × 10^5^ PFUs. The inoculation solution was then replaced by DMEM high glucose supplemented with 2% FBS, 1% 100X L-Glutamine, 1% P/S and 0.2 µg/ml TPCK-trypsin. Supernatants were taken 1, 16 and 24 h after infection and titrated on MDCK cells by a standard plaque assay to determine the infectious viral yield. For comparative growth kinetics of Pan/99(H3N2) on 2D cultured A549 cell monolayers, 2.5 × 10^5^ cells/well were infected at an MOI of 1. Cells were washed twice with 1x HBSS, inoculated with Pan/99(H3N2) for 45 min at room temperature and treated like described for 3D printed constructs.

### Statistical analysis

Statistical evaluation of experiments was performed using the student’s t-test (GraphPad Prism 6, GraphPad Software, Inc., La Jolla CA, USA). Each set of cell-laden experiments was repeated three times. Data are represented as mean ± SEM, p-values are considered significant by *p ≤ 0.05; **p ≤ 0.01; ***p ≤ 0.001.
